# Epigenetic Biomarkers in Cancer

**DOI:** 10.1155/2018/4987103

**Published:** 2018-02-20

**Authors:** Yuen Yee Cheng, Hong Chuan Jin, Michael W. Y. Chan, Wai Kit Chu, Michael Grusch

**Affiliations:** ^1^Asbestos Diseases Research Institute, The University of Sydney, Sydney, NSW, Australia; ^2^Biomedical Research Centre, Sir Run Run Shaw Hospital, Zhejiang University, Zhejiang, China; ^3^Department of Life Science, National Chung Cheng University, Chiayi, Taiwan; ^4^Department of Ophthalmology and Visual Sciences, The Chinese University of Hong Kong, Sha Tin, Hong Kong; ^5^Institute of Cancer Research, Medical University of Vienna, Vienna, Austria

We are pleased to announce the publication of this special issue focusing on epigenetic biomarkers in cancer. Epigenetics has gained the interest of researchers from all over the world; a total of 28 articles exist in this field that includes reviews and original research articles submitted for review. Among them, our editorial team, consisting of five prominent researchers in this field has selected four reviews and four original research articles for publication in this special issue. The reviews broadly cover the topics of DNA methylation, long noncoding RNAs, and single nucleotide polymorphisms, and the original research articles focus on DNA methylation, noncoding centromere RNAs, and mitochondria DNA. The selected articles highlight the progress in the field but suggest that there is also still a lack of translational linkage between epigenetic biomarker discoveries and their clinical application.

Exciting developments are occurring in the areas of early cancer detection, biomarker-based treatment selection, monitoring of disease response to treatment and early detection of recurrence. This is because research efforts are beginning to tap the wealth of significant and disease-driving information in DNA methylation and long noncoding RNA and in the complex and disease-influencing area of epigenetic modifications. In this special edition, we survey these exciting developments that are documented in review articles and novel experimental research results in specific, focused areas. Y. Y. Cheng et al. present novel findings suggesting DNA methylation as a novel noninvasive diagnostic marker for mesothelioma. Mesothelioma is a deadly cancer that requires ongoing efforts to understand its molecular drivers and find any effective molecular-based treatments. This novel research represents sorely needed progress in the molecular understanding of mesothelioma. C. Leygo et al. review the current state of research into the detection of epigenetic biomarkers of cancer using noninvasive samples from patients—an area that is hoped will soon revolutionise cancer detection and monitoring of response to therapy. D. Y. L. Chan et al. present cutting-edge results from live cell imaging showing the effects of transcribed noncoding RNA centromeric satellites in generating aberrant chromosome segregation during cell division—a valuable contribution in the significantly influential yet little-studied area of noncoding RNA. L. Bolha et al. review the research into the use of long noncoding RNAs (lncRNAs) from noninvasive body fluids as cancer biomarkers. This is an area of significant potential as we discover the richness of tissue and disease-specific information contained in lncRNAs and their ability for detection in sick patients by noninvasive means. In a focused, detailed study, S. Li et al. measure the prevalence of specific germline SNPs in a gene-influencing immune system interaction and their relationships to nasopharyngeal cancer susceptibility. G. M. Dexheimer et al. have carried out an extensive literature review of detection of epigenetic aberration in acute myeloid leukemia and myelodysplastic syndrome. This is a disease where aberrant DNA is highly accessible and thus ripe for exploitation in our efforts to detect disease at earlier stages that are more successfully treated and to follow disease response and progression so as to more successfully modify treatments. J. Wu et al. use machine learning on TCGA methylation data to discover new biomarkers of lymph node metastasis in stomach cancer, having valuable predictive power for stomach cancer recurrence and treatment management for this fifth most common cancer in the world. H. Shuwen et al. carry out a review of current novel research that uses mitochondrial data—copy number variations, displacement loop mutations, variant burden, and microsatellite instability—to find more sensitive and accurate biomarkers in colorectal cancer. This is a timely review given controversies in this novel area of investigation.

The field of epigenetic regulation is an attractive area of research which was first studied in the 1970s and quickly gained global interest. Epigenetic regulation is involved in almost all developmental processes of mammalian cells from fertilisation, implantation, and differentiation during embryonic development to aging and carcinogenesis. Different stages of development are, thus, characterized by differences in epigenetic signatures, and variations in epigenetic patterns may also be associated with specific stages of disease. Carcinogenesis is a multistep process, and we believe that detection of changes in epigenetic profiles can be exploited to differentiate not only between different types of malignancies but also between different stages of cancer progression. Epigenetic biomarkers thus hold great promise to become more conclusive diagnostic and prognostic biomarkers for different cancers. DNA methylation is the most studied area of epigenetics, yet there are still only few methylation markers currently used in the clinical setting. We anticipate, however, that with the development of new technologies and due to their stability in cells and the circulation, DNA methylation biomarkers will play a prominent role in the near future. Further research is required in this field to ensure the widespread application of DNA methylation markers in clinical settings.

In conclusion, epigenetic regulation plays a major role in cancer formation, and analysis of epigenetic biomarkers has great potential to become clinically relevant. More studies are required to further develop and evaluate the clinical application of epigenetic biomarkers. This special issue brings together reviews and original articles focusing on both basic and translational research in the field of epigenetic biomarkers. We are confident that this special issue will advance the understanding and stimulate further research of epigenetic biomarkers in cancer.

